# The impact of early and late childcare experience on cognitive functions

**DOI:** 10.1192/j.eurpsy.2022.1573

**Published:** 2022-09-01

**Authors:** A.Z. Békefi, J. Kárpáti, J. Futó

**Affiliations:** Eötvös Loránd University, Department Of Developmental And Clinical Child Psychology, Budapest, Hungary

**Keywords:** childcare, Executive functions, institutionalisation, attachment security

## Abstract

**Introduction:**

Previous studies have found long lasting cognitive delays among children with early childcare experience, especially institutionalised experience. However, little is known about institutions’ effect in late childhood.

**Objectives:**

Our goal is to identify the characteristics of cognitive functions in connection to attachment related anxiety among adopted children and children living in institutional care.

**Methods:**

The participants’ (N=68, Mage=14.20, 29 boys and 39 girls) cognitive functions were measured with the following tests: Rey15 Memory Task, Knock And Tap Task, Simon Says Test, Verbal Fluency Task, D-KEFS 20 Questions Test. Participants completed two questionnaires: the Family Affluence Scale and the Experiences In Close Relationships Revised Scale. The results from the adopted children (N=19) and children living in institutional care (N=18) were compared to the matched control group: children living with their biological parents (N=31).

**Results:**

Children living in institutional care did not differ significantly from their (SES-based) matched controls. Children adopted after the age of 2 years (N=7, M =56,57month) and the low SES control group (N=14) differed from the high SES control group on tests of attention *(Verbal Fluency Task*, *Mhigh.c.*=212.50, *Mad.aft.2=*193.50, *U=*59.50*, z=*-2.62*, p=*0.009*)* and verbal memory *(Rey15, Mhigh.c.*=17.94*, Mad.aft.2*=9.18, *U=*35.00, *z=*-2.79, *p=*0.005*).* Children adopted before the age of 2 years differed from the high SES control as well, in inhibition *(Simon Says Test, Mhigh.c.=*12.26*, Mad.bef.2*=18.88, *U*=55.55, *z* =-2.23*, p*=0.026*).*

**Conclusions:**

Our findings suggest that only in the early years is child protection experience associated with long-lasting cognitive delays and attachment related anxiety.

**Disclosure:**

No significant relationships.

